# The Effect of Using Hypotension Prediction Index to Reduce Intraoperative Hypotension in Elective Cesarean Sections: A Randomized Controlled Trial

**DOI:** 10.3390/medicina62081486

**Published:** 2026-08-01

**Authors:** Nadhirah Abd Halim, Azlina Masdar, Syarifah Noor Nazihah Sayed Masri, Iskandar Khalid, Maryam Budiman, Saw Kian Cheah

**Affiliations:** 1 Department of Anesthesiology and Intensive Care, Faculty of Medicine, Universiti Kebangsaan Malaysia, Jalan Yaacob Latif, Bandar Tun Razak, Cheras, Kuala Lumpur 56000, Malaysia; naddyhalim90@gmail.com (N.A.H.); cheah.saw.kian@hctm.ukm.edu.my (S.K.C.); 2 Department of Anesthesiology and Intensive Care, Hospital Canselor Tuanku Muhriz, Jalan Yaacob Latif, Bandar Tun Razak, Cheras, Kuala Lumpur 56000, Malaysia; nazihah@hctm.ukm.edu.my (S.N.N.S.M.); iskandar.hctm@ukm.edu.my (I.K.); maryam.budiman@hctm.ukm.edu.my (M.B.)

**Keywords:** hypotension, cesarean section, spinal anesthesia, PONV, non-invasive monitoring, obstetric complication, fetal outcome

## Abstract

*Background and Objectives:* The Hypotension Prediction Index (HPI) is a predictive algorithm that enables proactive management of intraoperative hypotension (IOH) and has been integrated into finger cuff devices for practical use in obstetric settings. This study aims to evaluate the effectiveness of HPI in reducing IOH during elective cesarean sections (CS). *Materials and Methods:* This randomized controlled trial enrolled parturients undergoing elective CS under spinal anesthesia. The parturients were randomized to either the HPI or the control group using non-invasive blood pressure (NIBP) monitoring. The primary outcome was the time-weighted average mean arterial pressure below 65 mmHg (TWA-MAP < 65 mmHg). Secondary outcomes included maternal nausea and vomiting, intraoperative fluid and vasopressor requirements, incidences of hypertension and bradycardia, estimated blood loss, duration of high-dependency unit admission, fetal Apgar scores, umbilical cord pH, and length of neonatal intensive care unit (NICU) stay. *Results:* A total of 100 parturients were enrolled in the study, with 96 included in the analysis. Baseline demographic characteristics were similar between the groups. The TWA-MAP < 65 mmHg was significantly lower in the HPI group (0.24 [0, 0.70] vs. 1.67 [0.71, 2.57] mmHg; *p* < 0.001). The incidence of intraoperative and postoperative nausea and vomiting were significantly reduced in the HPI group (*p* < 0.01). Incidence of intraoperative hypertension and bradycardia, fetal Apgar scores, umbilical cord pH, and length of NICU stay did not differ between the two groups (*p* > 0.05). *Conclusions:* HPI-guided management was associated with a reduction in hypotension burden and perioperative nausea and vomiting.

## 1. Introduction

Intraoperative hypotension (IOH) leads to perioperative morbidity and mortality. It carries risks of acute kidney injury, myocardial injury, and stroke, leading to poor patient outcomes [1,2,3,4]. The definition of IOH varies widely [5]. One of the generally accepted definitions of IOH is a mean arterial pressure (MAP) of 65 mmHg and below for at least 1 min, as MAP of more than 65 mmHg is associated with adequate tissue perfusion [1,6]. Prolonged IOH in cesarean sections (CS) is associated with a higher incidence of maternal nausea and vomiting, and in severe cases, may result in cardiovascular collapse [7,8]. The effects on the fetus include fetal acidemia, lower Apgar scores and increased requirements for neonatal resuscitation [8,9].

A study by Zwane et al. found that 49% of patients experienced IOH throughout CS [10]. This high incidence has led to extensive research on methods to prevent IOH by employing different practices in terms of fluid administration, prophylactic vasopressor using phenylephrine infusion, maternal position and weight- and height-adjusted spinal anesthesia volumes [11,12,13,14,15,16]. The Royal College of Anesthetists, United Kingdom, recommends using at least an oscillometric non-invasive blood pressure (NIBP) monitoring throughout the CS, and this is practiced worldwide [17]. While standard NIBP creates temporal “blind spots” between readings, continuous non-invasive arterial systems offer real-time tracking. However, the latter remains reactive, as it provides treatment only after hypotension has occurred. Consequently, extensive research has focused on strategies to mitigate IOH, with increasing emphasis on the integration of proactive hemodynamic management protocols guided by machine learning predictive algorithms [18,19,20].

The Hypotension Prediction Index (HPI), developed by Edwards Lifesciences, Irvine, United States of America (USA), utilizes complex algorithms to detect subtle features in high-fidelity arterial pressure waveforms, generating a unitless index from 0 to 100 that indicates the probability of impending hypotension as the index increases [19,20,21]. An HPI of 85 and above is set as a threshold to intervene according to the algorithm [19,20,21]. This HPI threshold has a sensitivity and specificity of 92% and 92% at 5 min, 89% and 90% at 10 min, and 88% and 87% at 15 min before hypotension occurs [19]. Hence, it is reliable for predicting hypotension 5 to 15 min in advance, enabling the institution of treatment before the occurrence of hypotension. The Hypotension Prediction Index (HPI) has been shown to reduce IOH in various major non-cardiac surgeries [22,23,24,25,26].

The development of the non-invasive Acumen IQ cuff bypasses the risks associated with invasive arterial lines, and it has been shown to be reliable and in agreement with radial artery blood pressure measurements [27,28]. Frassanito et al. found that the HPI was able to predict maternal hypotension during CS under spinal anesthesia, with an impressive sensitivity and specificity of 83% and 83% at 3 min, 97% and 97% at 2 min, and 100% and 100% at 1 min before it occurs [29].

However, there remains a critical need for prospective, randomized controlled evaluation of the clinical efficacy of intervening based on predictive algorithms, specifically in obstetric surgery such as CS. This study aims to determine whether proactive hemodynamic management utilizing the HPI algorithm via a non-invasive finger cuff significantly reduces IOH and its associated morbidities during elective CS, thus justifying a paradigm shift in obstetric anesthesia practice. We hypothesized that hemodynamic management guided by the HPI would reduce the intraoperative hypotensive burden during CS.

## 2. Materials and Methods

### 2.1. Study Design

This single-center, single-blinded, prospective randomized controlled trial was conducted at Hospital Canselor Tuanku Muhriz Universiti Kebangsaan Malaysia (HCTM UKM), Kuala Lumpur, between December 2024 and December 2025. It adhered to the Consolidated Standards of Reporting Trials (CONSORT) guidelines and was conducted following approval from the UKM Research Ethics Committee (JEP-2024-463) and registered at ClinicalTrials.gov (NCT06892665). The completed CONSORT checklist is provided as a Supplementary File S1. The aim was to determine whether HPI-guided hemodynamic management is effective in reducing IOH in elective CS under spinal anesthesia. 

### 2.2. Eligibility Criteria

Parturients aged 18–40 years scheduled for elective CS under spinal anesthesia, at term with a singleton pregnancy, were eligible for inclusion. Parturients were excluded if they were ASA III or above, had a body mass index of 40 kg/m^2^ or more, had an increased risk of developing peripartum hemorrhage, or were contraindicated for Acumen IQ cuff application, particularly due to cardiac arrhythmias and aortic regurgitation [30,31,32].

Parturients were dropped from the study if they required conversion to general anesthesia (GA), if the surgery was postponed after recruitment, if emergency CS was required, or if the parturient was uncontactable after 3 attempts at a phone call.

### 2.3. Randomization, Allocation and Blinding

Before the surgery, eligible participants were randomly assigned to two groups: one receiving proactive hemodynamic management guided by HPI (HPI group) and the other receiving standard hemodynamic management using NIBP (control group). Computer-generated randomization was conducted with a 1:1 allocation ratio using variable block sizes to ensure balanced allocation. To maintain allocation concealment, group assignments were placed in sequentially numbered, opaque, sealed envelopes, which were opened only after patient enrollment. A single-blinded study design was used, with participants unaware of the assignment. The anesthesiologist could not be blinded, as they needed to manage hemodynamics according to the study protocol. The postoperative outcome assessor was blinded to the allocation to reduce bias.

### 2.4. Enrollment Protocol

Standard monitoring, including a 3-lead continuous electrocardiogram, pulse oximetry, and NIBP in the upper arm, was applied to all parturients. The NIBP monitoring was performed every 1 min before and every 3 min after the baby was delivered, up to 90 min of surgery duration. After 90 min, data collection ceased. In addition to standard monitoring, all parturients had an Acumen IQ cuff placed on their third fingers, connected to the HemoSphere monitor equipped with the Acumen Analytic 2.0 software (Edwards Lifesciences, Irvine, CA, USA).

**Figure 1:**
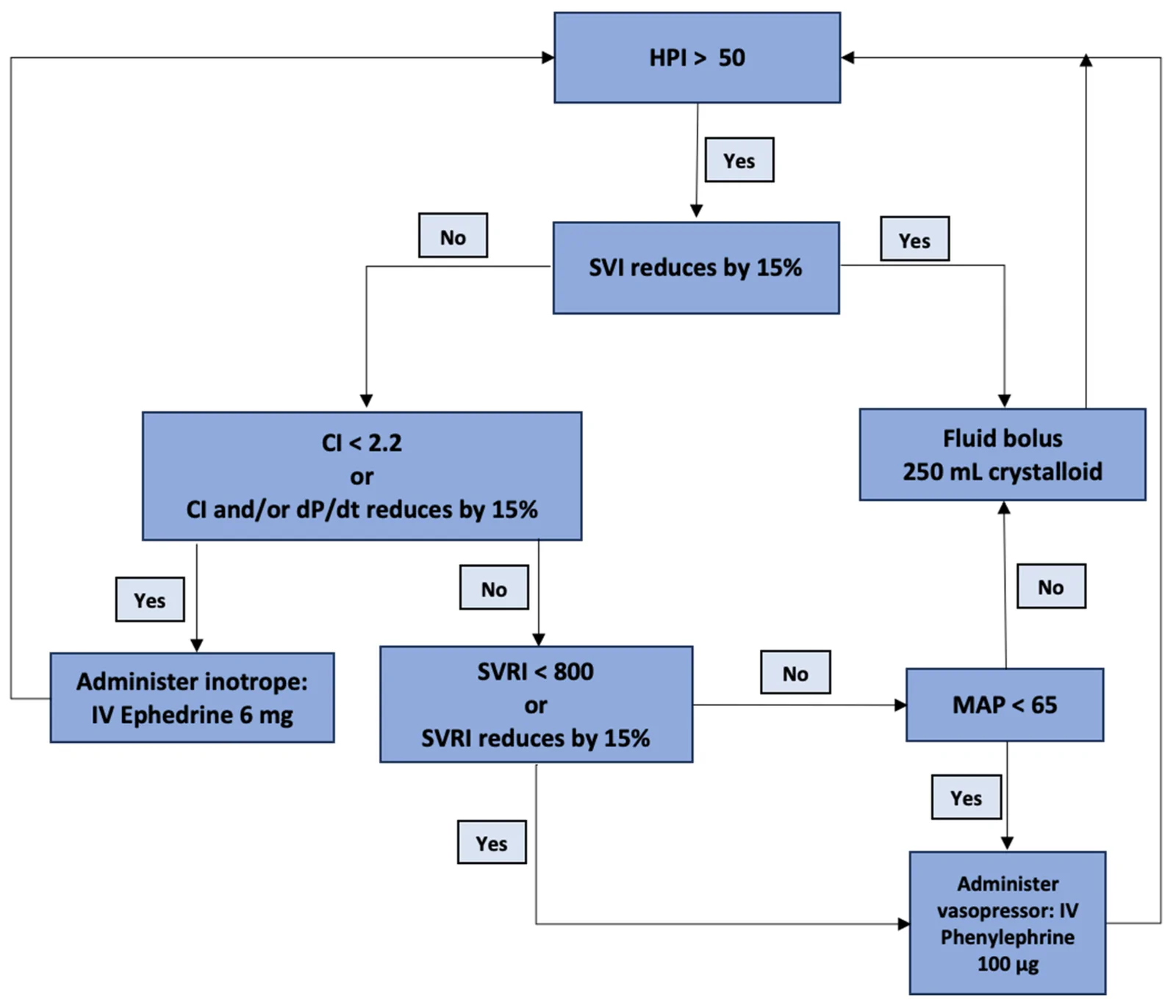
Algorithm for hemodynamic management in the HPI group, with HPI 50–84. HPI = Hypotension Prediction Index, SVI = Stroke Volume Index, dP/dt = change in pressure over change in time, CI = Cardiac Index, SVRI = Systemic Vascular Resistance Index, and MAP = mean arterial pressure. This algorithm was adapted from Guerra-Londono et al. [33].

**Figure 2:**
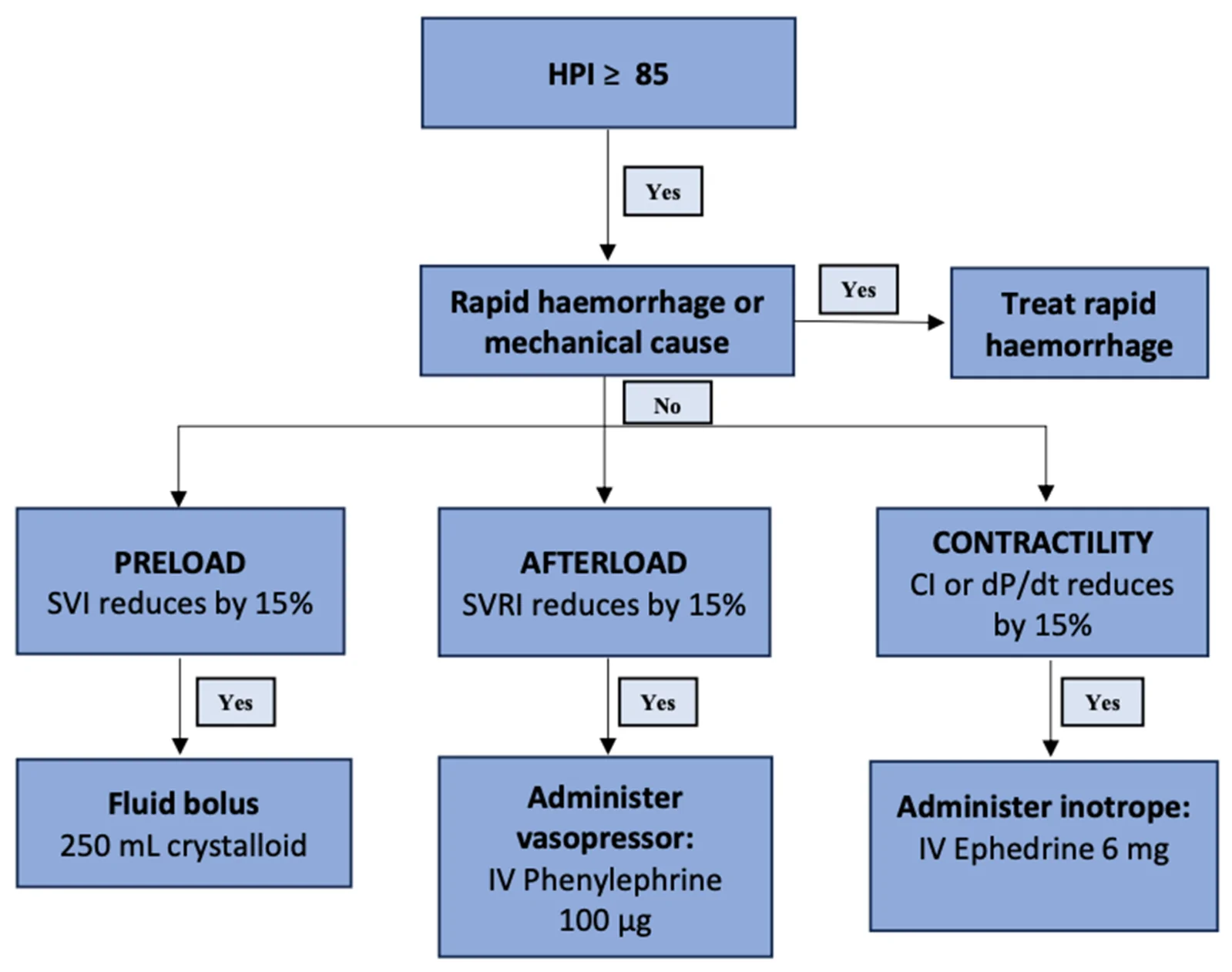
Algorithm for hemodynamic management in the HPI group, with HPI > 85. HPI = Hypotension Prediction Index, SVI = Stroke Volume Index, dP/dt = change in pressure over change in time, CI = Cardiac Index, SVRI = Systemic Vascular Resistance Index, MAP = mean arterial pressure. This algorithm was adapted from Maheshwari et al. [34].

In the HPI group, the HemoSphere monitor, displaying all hemodynamic parameters, was made visible for the anesthesiologist to act upon. The HPI audio-visual alarm was set to 85, as per the manufacturer’s setting. The management was HPI-based, following algorithms outlined in Figure 1 and Figure 2. As some studies suggested more proactive management using a lower threshold, our algorithms included intervention for when HPI increased between 50 and 84, and when it was more than 85. The anesthesiologist would have to assess for fluid responsiveness, vasodilatation, or reduced cardiac contractility, and manage accordingly.

**Figure 3:**
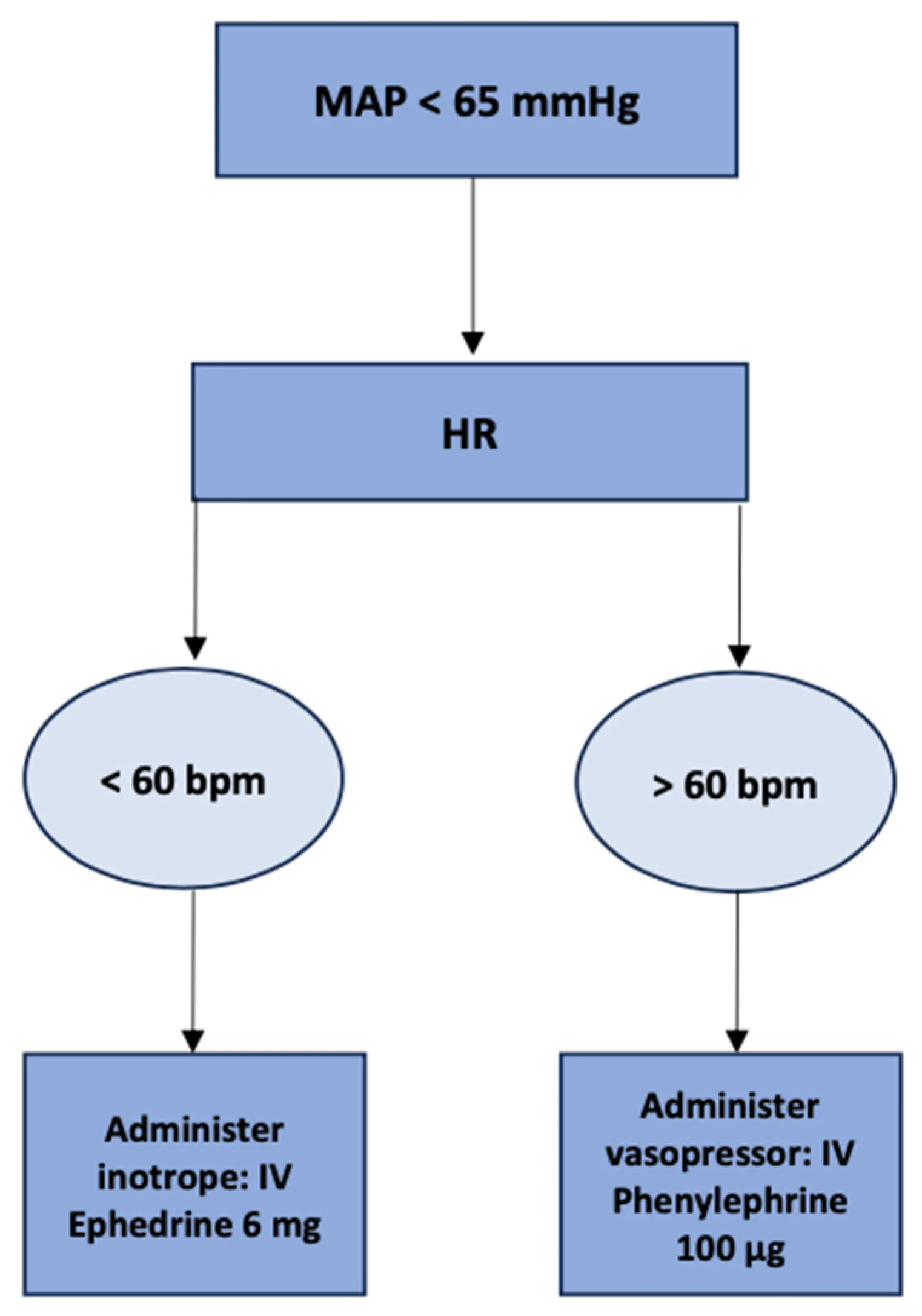
Algorithm for hemodynamic management in the control group.

In the control group, the HemoSphere monitor was concealed and the alarms silenced. Parturients in the control group were managed according to the standard protocol, outlined in Figure 3. If the MAP fell below 65 mmHg, either an IV phenylephrine or ephedrine bolus was administered, guided by the heart rate, until the MAP exceeded 65 mmHg. Rescue IV phenylephrine boluses were also administered if the parturient was tachycardic after spinal anesthesia, at the discretion of the attending anesthesiologist. Fluid administration was left to the discretion of the attending anesthesiologist.

### 2.5. Protocol Details

In the operating theatre, the parturients were positioned supine with a left lateral tilt. An 18-G intravenous cannula was secured. All parturients received a co-loading fluid of lactated Ringer’s solution at a rate of 15 mL/kg/hour. Spinal anesthesia was administered using a Pencan^®^ 27 G spinal needle (B.Braun, Melsungen, Germany) at the L3/L4 or L4/5 interspace in a sitting position by the anesthesiology medical officer assigned to the operating room. Local anesthesia infiltration with lignocaine 2% preceded spinal anesthesia. The spinal anesthesia solution consisted of intrathecal (IT) hyperbaric bupivacaine 0.5%, IT fentanyl 15 µg, and IT morphine 0.1 mg. The volume of hyperbaric bupivacaine 0.5% was dose-adjusted according to the parturient’s height and weight as per Siddiqui et al. [12]. After spinal anesthesia was administered, the parturient was repositioned supine with a 15-degree left lateral tilt. The onset of sensory block was assessed after 1 min using the pinprick method. Surgery commenced once the adequacy of the sensory block was established, targeting a sensory block up to the level of T4–T6.

After delivery, uterotonic agents (IV Oxytocin 5 IU slow bolus or IV carbetocin 100 µg) were administered. Additional medications, such as analgesics (IV/suppository paracetamol 1 g and suppository diclofenac 1 mg/kg) and anti-emetics (IV dexamethasone 8 mg and IV granisetron 1 mg), were administered if not contraindicated. The intravenous fluid regimen after delivery was left to the discretion of the attending anesthesiologist. Rescue drugs to manage hemodynamics, such as IV atropine 0.5–1 mg bolus for symptomatic bradycardia, were available for both groups.

Maternal nausea and vomiting, as well as neonatal outcomes such as umbilical cord pH from both the umbilical artery and vein and Apgar scores, were recorded.

Hemodynamic data were saved from the HemoSphere monitor from the start of surgery up to 90 min. This data included HPI, MAP, heart rate, systolic blood pressure (SBP), diastolic blood pressure (DBP), stroke volume variation (SVV), stroke volume index (SVI), cardiac index (CI), systemic vascular resistance index (SVRI), dynamic arterial elastance (Ea dyn), and change of pressure over change of time (dP/dt). The duration and severity of hypotension were demonstrated using a time-weighted average of MAP below 65 mmHg (TWA-MAP < 65 mmHg). The TWA-MAP < 65 mmHg was calculated using a formula adapted from previous studies on HPI [21,22,23,34,35,36,37,38]. The depth of hypotension represents the MAP decrease below hypotension threshold, calculated as (65 mmHg-measured MAP), expressed in mmHg.

TWA-MAP < 65 mmHg

= (the depth of hypotension of MAP below 65 mmHg × time spent below MAP of 65 mmHg (minutes))/(total duration of operation (minutes))

Post-operatively, the participants were followed up in the obstetric ward one and two days after surgery to assess maternal symptoms such as nausea and vomiting within 24 h, maternal satisfaction (using a Likert scale from 1 to 5), and other post-operative complications. Signs of overtreatment, such as intraoperative hypertension and bradycardia, were also recorded. Intraoperative hypertension was demonstrated using a time-weighted average of MAP greater than 100 mmHg (TWA-MAP > 100 mmHg) [36]. The magnitude of hypertension represents the increase of MAP above the hypertension threshold (MAP > 100 mmHg), calculated as (measured MAP-100), expressed in mmHg.

TWA-MAP > 100 mmHg

= (the magnitude of hypertension of MAP above 100 mmHg × time spent above MAP of 100 mmHg (minutes))/(total duration of operation (minutes))

Maternal and neonatal lengths of hospital stay were also recorded. Further follow-up after discharge was conducted via phone call 30 days after CS to gather information on post-operative complications such as surgical site infection and mortality.

### 2.6. Outcomes

The primary outcome of this study was to determine the duration and severity of hypotensive events, specifically reported as TWA-MAP < 65 mmHg, comparing the HPI group against the control group. Secondary outcomes included intraoperative hemodynamic outcomes, the quantity of fluids and blood products administered, blood loss and urine output, vasoactive agents, perioperative complications, and 30-day mortality. Neonatal outcomes analyzed were umbilical cord pH, Apgar scores, and duration of NICU stay.

### 2.7. Sample Size

The sample size calculation was based on a previous study by Yoshikawa et al., which reported a significant difference in TWA between the HPI and FloTrac (non-HPI) groups, with a mean difference of 0.47 mmHg and a pooled standard deviation of 0.80 [23]. The alpha value was set at 0.05, and the study power was 80%. Using the Snedecor and Cochran formula [39], and accounting for a 10% dropout rate, a total of 100 parturients were recruited for the study.

### 2.8. Statistical Analysis

All data analysis was performed using SPSS for Windows version 23.0. Results were presented as means ± standard deviations, medians, or frequencies where appropriate. Data normality was evaluated using the Shapiro–Wilk test. For between-group analysis, an independent t-test or Mann–Whitney U test was used for normally distributed continuous data and for not normally distributed data, respectively. Qualitative data analysis was done using the Pearson chi-squared test. A *p*-value < 0.05 was considered statistically significant. For the multivariable logistic regression model evaluating perioperative nausea and vomiting, candidate variables with a univariate entry threshold of *p* < 0.10 were included. The predictive performance of HPI for detecting impending IOH was evaluated using receiver operating characteristic (ROC) analysis. Missing data were managed via complete-case analysis; participants with complete primary outcome data were included in the final analysis.

## 3. Results

**Figure 4:**
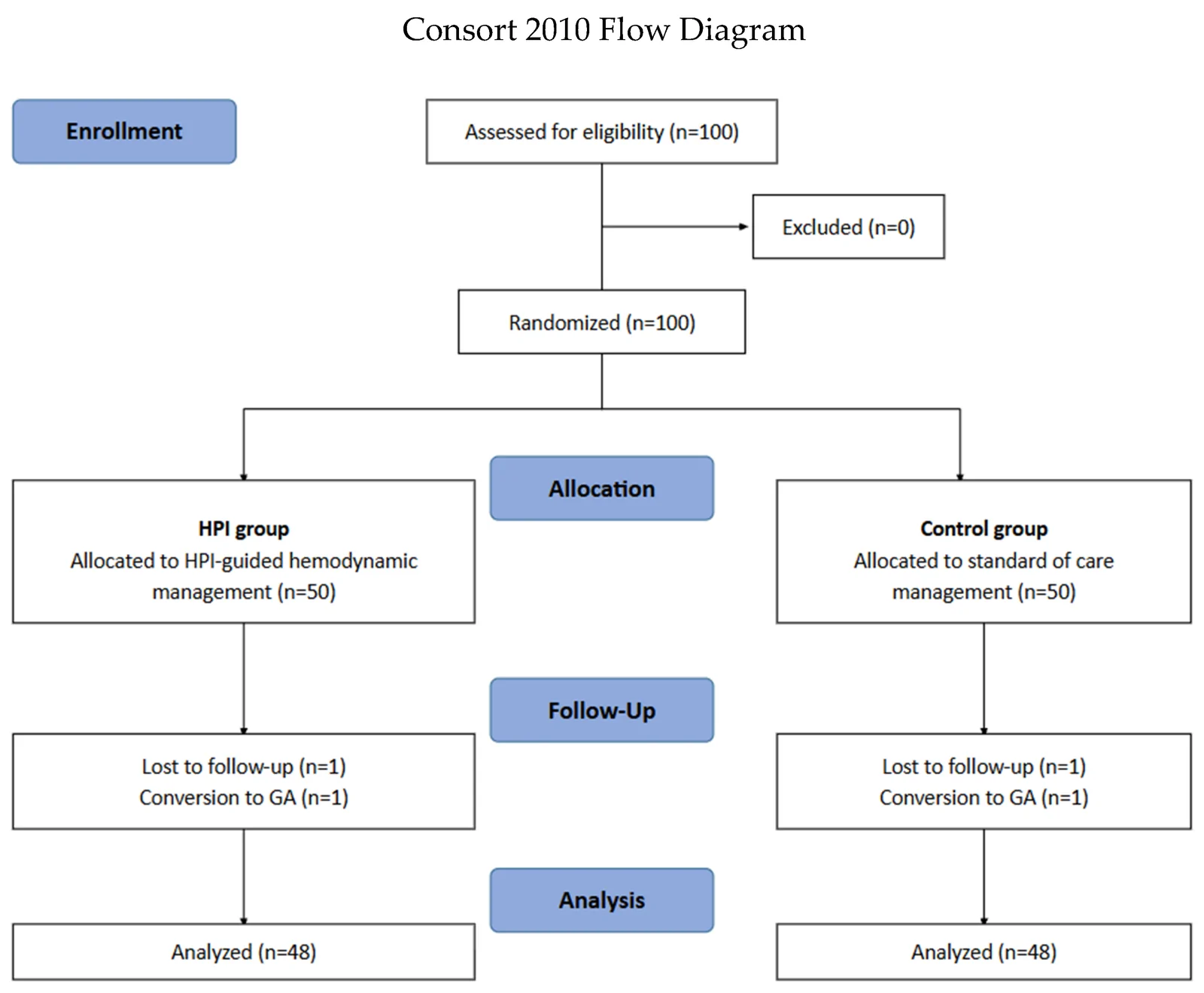
Flow diagram of study patients according to the CONSORT for randomized controlled trials.

A total of 100 parturients were randomized, with 96 parturients included in the final analysis. Four parturients were excluded from the final analysis due to conversion to GA and loss to follow-up (Figure 4).

Baseline demographics, comorbidities, preoperative hemoglobin, baseline hemodynamic parameters and duration of surgery were comparable between the HPI and control groups, as outlined in Table 1.

**Figure 5:**
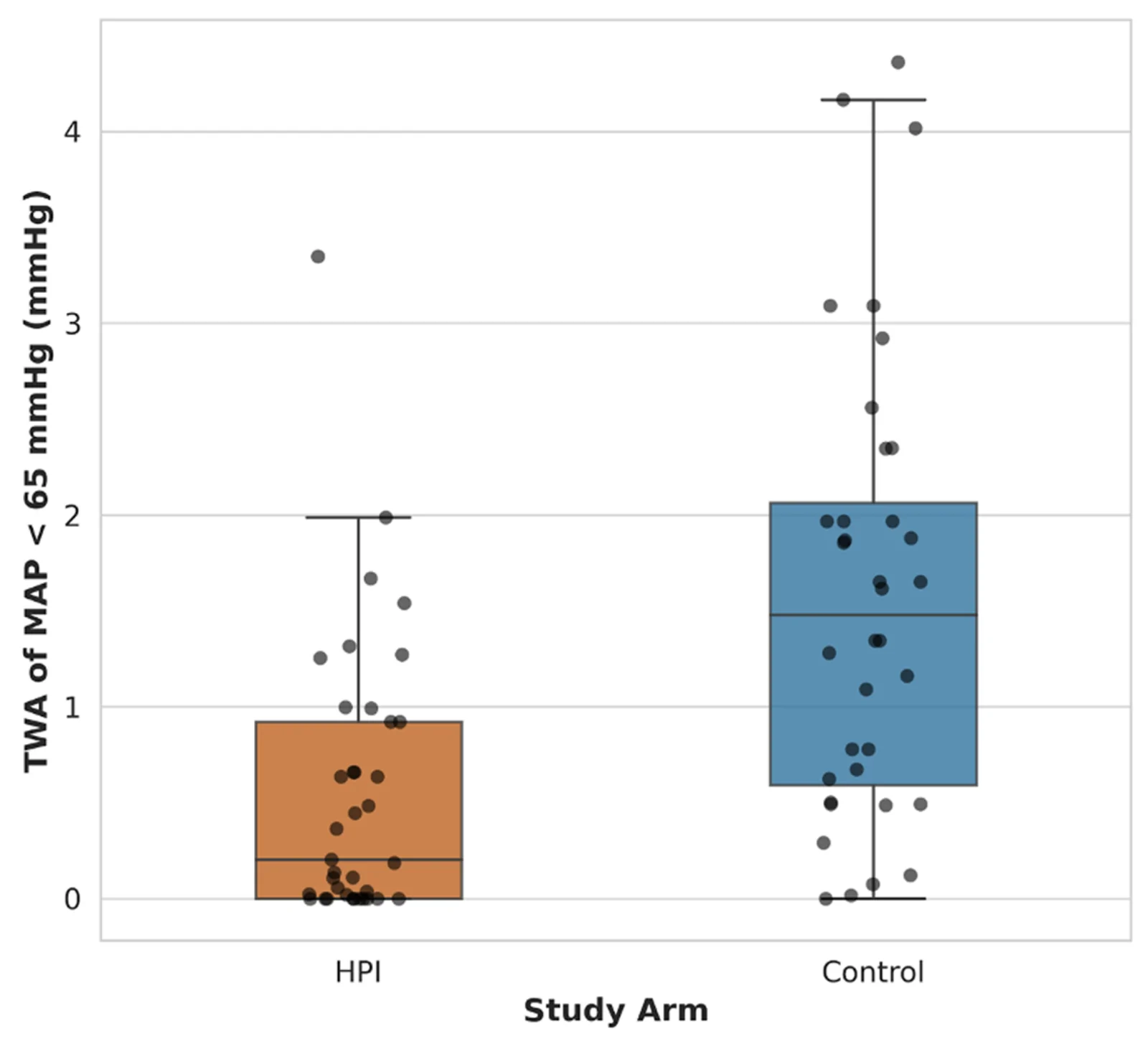
Box plot comparison of TWA-MAP < 65 mmHg (*p*-value < 0.001).

Intraoperative hypotension (IOH), represented as TWA-MAP < 65 mmHg, was significantly reduced in the HPI group, as outlined in Figure 5 and Table 2. This marked reduction in cumulative hypotension burden indicates that IOH was successfully mitigated with proactive hemodynamic management.

The HPI group demonstrated a significantly higher dose of phenylephrine administered, as shown in Table 3. Despite this, proactive hemodynamic management did not increase the risk of overtreatment. Intraoperative hypertension, represented as TWA-MAP > 100 mmHg, was negligible and comparable between the groups, and there were no observed episodes of intraoperative bradycardia in either cohort, as shown in Table 2.

Along with a significant reduction in IOH, parturients in the HPI group experienced significantly less intraoperative nausea and vomiting. Similar results were found during the postoperative period. This demonstrates that minimizing cumulative hypotension burden provides a clinical benefit by preserving maternal comfort and reducing emetic symptoms. The length of hospital and HDU stay, maternal satisfaction, surgical site infection and 30-day mortality were comparable between groups, as shown in Table 4.

A logistic regression analysis was conducted to evaluate whether the HPI-guided algorithm was able to reduce the odds of both intraoperative and postoperative nausea and vomiting, as shown in Table 5. There was a clinical trend towards reduced odds. However, it did not reach statistical significance in the logistic regression model.

Neonatal outcomes, including Apgar scores at 1 and 5 min, umbilical arterial and venous cord pH, and length of hospital and NICU stay, were similar between the two groups, as shown in Table 6.

**Figure 6:**
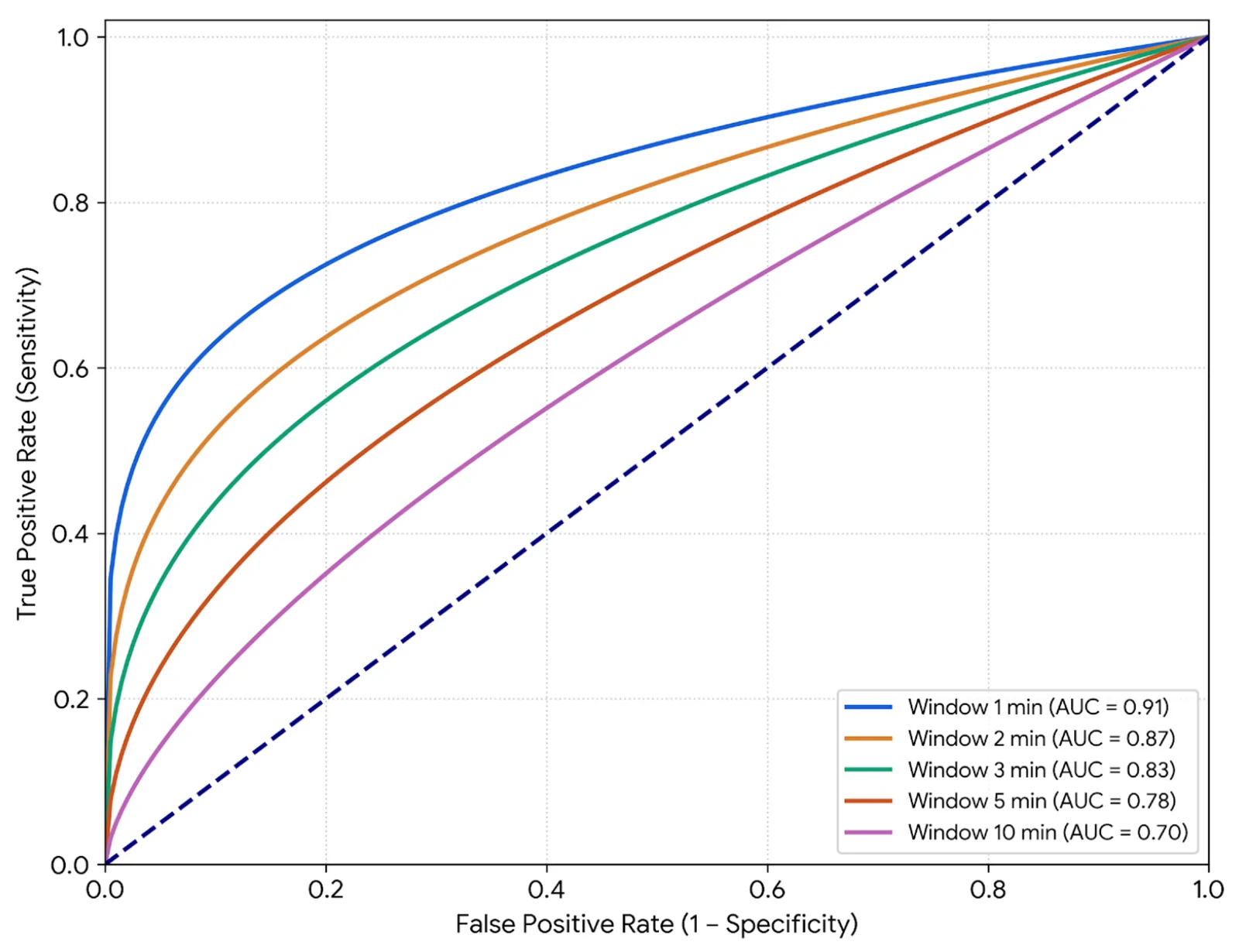
Predictive ROC curves for HPI predicting hypotension. ROC = Receiver Operating Characteristic.

Similar to Frassanito et al., we also found that the receiver operating characteristic (ROC) analysis demonstrated that HPI is reliable in predicting hypotension in this group of patients [29]. The predictive accuracy remains high even at 10 min, as shown in Figure 6.

## 4. Discussion

This randomized controlled trial demonstrated a significant reduction in IOH during elective CS when hemodynamics were managed proactively with the HPI algorithm.

When comparing our study to other trials utilizing non-invasive Acumen or ClearSight technology, a critical question arises: Was the observed improvement in IOH primarily due to the HPI predictive algorithm, or simply due to the availability of beat-to-beat arterial waveform [18,19,20,21,22,23,24,25,26]? Vos and Scheeren highlighted that intermittent NIBP monitoring leaves critical “blind spots” that fail to detect short yet harmful periods of IOH, whereas continuous monitoring allows for more efficient reactive treatment [20]. However, even with continuous monitoring, treatment is instituted as a reactive treatment once hypotension has already occurred, leaving a therapeutic lag before the vasopressor can work to restore normal vascular tone and normotension [20]. The HPI algorithm overcomes this physiological lag by predicting hypotension up to 15 min before it occurs, allowing for proactive treatment before the hypotensive episode [19].

The clinical impact of this proactive approach is most evident in the trend of vasopressor use. The HPI group demonstrated a significantly higher dose of phenylephrine administered compared to the control group. This is not an indication of a management failure, but rather a shift from “rescue” boluses to “stability” dosing. In standard reactive management, phenylephrine is administered as a rescue medication to correct IOH. Conversely, in the HPI group, phenylephrine was administered while the patient was still normotensive, based on the probability of impending IOH. This pre-emptive administration ensures that vascular tone is maintained before spinal anesthesia-induced vasodilation manifests as hypotension.

A systematic review by Singh et al. noted that continuous infusion of α-agonists with mild β-activity, like noradrenaline, is often preferable to mixed agonists like ephedrine due to a more favorable hemodynamic profile [40]. It is also worth noting that a meta-analysis on preeclamptic women by Ahmed et al. reported superior maternal safety when noradrenaline was used compared to phenylephrine, due to a lower incidence of bradycardia [41]. However, in our trial, we found that despite receiving more than double the dose of phenylephrine, guided by the predictive HPI algorithm, the incidence of bradycardia was 0%. We hypothesize that when given in titrated doses, guided by HPI and other hemodynamic parameters, phenylephrine remains a safe vasoactive agent for parturients undergoing CS.

This optimization of maternal hemodynamics throughout CS translated to a reduction in both intraoperative and postoperative nausea and vomiting. This confirms that preventing even moderate cumulative hypotension exposure is critical to maintaining visceral perfusion and mitigating triggers of nausea and vomiting perioperatively. Although maternal satisfaction scores did not reach statistical significance, we hypothesize that the overall maternal experience is enhanced by the improved comfort provided by proactive hemodynamic management.

A study by Shih et al. comparing the effectiveness of HPI in reducing IOH in CS with continuous non-invasive arterial pressure and NIBP confirmed the synergistic effect of HPI in reducing IOH [42]. They reported a lower TWA-MAP < 65 mmHg in their HPI group compared to ours; such variation likely stems from a higher baseline hypotension burden in our control group [42]. Moreover, they adopted a different protocol utilizing intermittent noradrenaline boluses instead of phenylephrine and ephedrine [42]. Nonetheless, the study confirmed our secondary findings, whereby parturients managed with HPI experienced a significantly lower incidence of perioperative nausea and vomiting [42].

The physiological link between IOH and emesis is primarily driven by acute hypoperfusion. IOH leads to transient cerebral hypoperfusion, which can directly stimulate the chemoreceptor trigger zone [43]. Concurrently, a precipitous drop in MAP compromises splanchnic perfusion, leading to the release of serotonin from enterochromaffin cells during intestinal ischemia [43]. This translates to transmission of potent emetogenic signals via vagal afferents [43]. The reduction in nausea and vomiting incidence noted in the HPI group continued into the postoperative phase. This enduring advantage arises because proactive hemodynamic management averts the onset of the ischemic cascade. By maintaining more precise hemodynamic control, HPI guidance effectively prevents these physiological triggers, thus mitigating both early and delayed postoperative nausea and vomiting.

A common concern with proactive vasopressor use is the risk of overtreatment, which can lead to either hypertension or bradycardia. However, we found that the incidence of hypertension and bradycardia showed no significant difference between the two groups.

While IOH was significantly lower with HPI guidance, other neonatal outcomes (Apgar scores, umbilical cord pH, hospital and NICU stay) did not show any significant differences. This is commonly observed in studies involving healthy elective cesarean sections, where the robust fetus and uteroplacental unit possess the physiological capacity to withstand temporarily and reactively managed hypotension without showing any substantial effects [41].

A recognized limitation of this study is the inability to fully isolate the independent effects of the HPI algorithm from those of continuous non-invasive arterial pressure monitoring. The method was adopted to compare HPI with standard management. Future research with continuous arterial pressure monitoring as one of the study arms would help further delineate these parallel therapeutic effects. Additionally, this study was a single-blinded study, whereby blinding the attending anesthesiologist was unfeasible, as they needed to manage hemodynamics according to the study protocol. The study may not be sufficiently powered to detect subtle differences in neonatal outcomes. Future multicenter trials could focus on high-risk pregnancies where the benefits to the fetus might be more pronounced.

## 5. Conclusions

The study provides evidence that HPI-guided management significantly reduces the burden of IOH and improves the maternal perioperative experience during elective CS.

## Figures and Tables

**Table 1:** Baseline demographics, comorbidities, preoperative hemoglobin, baseline hemodynamic parameters and duration of surgery between the HPI and control groups.

	HPI Group (*n* = 48)	Control Group (*n* = 48)	*p*-Value
Baseline characteristics			
Age (years)	34.31 ± 4.04	34.96 ± 3.70	0.416 ^a^
BMI (kg/m^2^)	30.49 ± 5.16	31.68 ± 4.11	0.212 ^a^
Gravidity (*n*)	2.54 ± 1.13	2.77 ± 1.24	0.347 ^a^
Parity (*n*)	1.26 ± 0.96	1.48 ± 1.03	0.347 ^a^
Gestational age (weeks)	38.15 ± 0.53	38.23 ± 0.81	0.553 ^a^
Number of previous CS (*n*)	0.79 ± 0.95	1.15 ± 0.92	0.665 ^a^
Comorbidities			
Hypertension, *n* (%)	2 (4.2)	2 (4.2)	1.000 ^b^
Diabetes mellitus, *n* (%)	14 (29.2)	14 (29.2)	1.000 ^b^
Anemia, *n* (%)	10 (20.8)	16 (33.3)	0.168 ^b^
Asthma, *n* (%)	3 (6.3)	1 (2.1)	0.307 ^b^
OSA, *n* (%)	1 (2.1)	0 (0)	0.315 ^b^
Baseline hemoglobin, hemodynamic parameters and duration			
Baseline Hb (g/dL)	11.5 [10.6, 12.4]	11.6 [10.3, 12.5]	0.871 ^c^
Baseline MAP (mmHg)	83.17 ± 12.49	84.13 ± 12.51	0.708 ^a^
Baseline HPI	35.88 ± 26.75	34.48 ± 25.40	0.794 ^a^
Baseline SVI (mL/b/m^2^)	43.02 ± 6.15	44.38 ± 7.38	0.571 ^a^
Baseline SVV (%)	11.5 ± 4.6	12.1 ± 4.1	0.463 ^a^
Baseline dP/dt (mmHg/s)	974.98 ± 190.36	903.29 ± 267.16	0.133 ^a^
Baseline CI (L/min/m^2^)	3.72 ± 0.92	3.59 ± 1.06	0.513 ^a^
Baseline SVRI (dynes·cm^−5^·m^2^)	2059.60 ± 219.23	1805.46 ± 212.15	0.525 ^a^
Baseline Ea dyn	1.41 ± 0.46	1.50 ± 0.53	0.401 ^a^
Duration of surgery (min)	82.65 ± 21.41	80.18 ± 21.68	0.311 ^a^

BMI, body mass index; CS, cesarean section; OSA, obstructive sleep apnea; Hb, hemoglobin; MAP, mean arterial pressure; HPI, Hypotension Prediction Index; SVI, Stroke Volume Index; SVV, stroke volume variation; dP/dt, change in pressure over change in time; CI, Cardiac Index; SVRI, Systemic Vascular Resistance Index; Ea dyn, dynamic arterial elastance. Data were expressed as mean + SD, median [25th percentile-75th percentile] or n (%) as appropriate. ^a^ Independent sample *t* test; ^b^ Pearson chi-squared test; ^c^ Mann–Whitney U test.

**Table 2:** Hemodynamic outcomes.

Hemodynamic Outcomes	HPI Group (*n* = 48)	Control Group (*n* = 48)	*p*-Value
TWA-MAP < 65 (mmHg)	0.24 [0, 0.70]	1.67 [0.71, 2.57]	<0.001 ^a^
Duration of hypotension (minutes)	2.0 [0, 5.8]	13.3 [5.4, 19.9]	<0.001 ^a^
Percentage of time in hypotension (%)	2.40 [0, 7.90]	16.66 [8.36, 32.12]	<0.001 ^a^
AUT MAP < 65 (mmHg.min)	17.00 [0, 54.32]	119.67 [45.67, 183.00]	<0.001 ^a^
Incidence of MAP < 50 mmHg, *n* (%)	8 (16.7)	18 (37.5)	0.002 ^b^
Lowest MAP (mmHg)	55.69 ± 9.32	45.40 ± 8.80	<0.001 ^c^
TWA-MAP > 100 (mmHg)	0 [0, 0.27]	0 [0, 0.15]	0.591 ^a^
Incidence of bradycardia, *n* (%)	0(0)	0(0)	0.486 ^b^

TWA, time-weighted average; MAP, mean arterial pressure; AUT, area under threshold. Data were expressed as mean + SD, median [25th percentile-75th percentile], or n (%) as appropriate. ^a^ Mann–Whitney U test; ^b^ Pearson chi-squared test; ^c^ Independent sample *t* test.

**Table 3:** Intraoperative outcomes.

Intraoperative Outcomes	HPI Group (*n* = 48)	Control Group (*n* = 48)	*p*-Value
EBL (mL)	469.79 ± 368.69	425.00 ± 294.27	0.512 ^a^
Blood transfusion, *n* (%)	2 (4.2)	1 (2.1)	0.557 ^b^
Total fluids (mL)	1342.71 ± 488.05	1292.71 ± 503.39	0.622 ^a^
Urine output (mL)	300 [250, 450]	300 [250, 425]	0.608 ^c^
Phenylephrine (µg)	1400 ± 620	620 ± 480	<0.001 ^a^
Ephedrine (mg)	0 [0, 6]	3.15 [0, 7.5]	0.740 ^c^

EBL: Estimated blood loss. Data were expressed as mean + SD, median [25th percentile-75th percentile], or n (%) as appropriate. ^a^ Independent sample t test; ^b^ Pearson chi-squared test; ^c^ Mann–Whitney U test.

**Table 4:** Maternal outcomes.

Maternal Outcomes	HPI Group (*n* = 48)	Control Group (*n* = 48)	*p*-Value
Intraoperative nausea, *n* (%)	3 (6.3)	24 (50)	<0.001 ^a^
Intraoperative vomiting, *n* (%)	2 (4.2)	13 (27.1)	0.002 ^a^
Postoperative nausea, *n* (%)	3 (6.3)	23 (47.9)	<0.001 ^a^
Postoperative vomiting, *n* (%)	2 (4.2)	11 (22.9)	0.007 ^a^
Length of stay (days)	4 [4, 4]	4 [4, 5]	0.577 ^b^
Length of HDU stay (hours)	0 [0, 0]	0 [0, 14.25]	0.823 ^b^
Maternal satisfaction (Likert scale)	4.96 ± 0.20	4.85 ± 0.36	0.082 ^c^
Surgical site infection, *n* (%)	0 (0)	0 (0)	1.000 ^a^
30-day mortality, *n* (%)	0 (0)	0 (0)	1.000 ^a^

HDU: High dependency unit. Data were expressed as mean + SD, median [25th percentile-75th percentile] or n (%) as appropriate. ^a^ Pearson chi-squared test; ^b^ Mann–Whitney U test; ^c^ Independent sample *t* test.

**Table 5:** Logistic regression analysis for perioperative nausea and vomiting.

	OR	95% CI	*p*-Value
IONV	0.56	0.22–1.44	0.230 ^a^
PONV	0.58	0.23–1.43	0.235 ^a^

IONV: Intraoperative nausea and vomiting; PONV: Postoperative nausea and vomiting. ^a^ Wald test.

**Table 6:** Neonatal outcomes.

Neonatal Outcomes	HPI Group (*n* = 48)	Control Group (*n* = 48)	*p*-Value
Apgar score at 1 min	9 [9, 9]	9 [9, 9]	0.268 ^a^
Apgar score at 5 min	10 [10, 10]	10 [10, 10]	0.435 ^a^
Umbilical arterial cord pH	7.272 ± 0.058	7.278 ± 0.072	0.742 ^b^
Umbilical venous cord pH	7.313 ± 0.063	7.300 ± 0.056	0.467 ^b^
Length of stay (days)	3 [3,3]	3 [3, 3.25]	0.283 ^a^
Length of NICU stay (h)	0 [0, 0]	0 [0, 25]	0.500 ^a^

NICU: Neonatal Intensive Care Unit. Data were expressed in mean + SD, median [25th percentile-75th percentile] or n (%) as appropriate. ^a^ Mann–Whitney U test; ^b^ Independent sample *t* test.

## Data Availability

All relevant data are within the paper and its supporting materials. The data is available from the corresponding author upon request.

## Supplementary Materials

The following supporting information can be downloaded at https://www.mdpi.com/article/10.3390/medicina62081486/s1, Supplementary File S1: The CONSORT checklist.

## Abbreviations

The following abbreviations are used in this manuscript:

CONSORT	Consolidated Standards of Reporting Trials
CS	cesarean section
CI	cardiac index
dP/dt	change of pressure over change of time
DBP	diastolic blood pressure
GA	general anesthesia
HPI	Hypotension Prediction Index
IOH	intraoperative hypotension
IT	intrathecal
MAP	mean arterial pressure
NIBP	non-invasive blood pressure
NICU	neonatal intensive care unit
ROC	Receiver Operating Characteristic
SVV	stroke volume variation
SVI	stroke volume index
SBP	systolic blood pressure
SVRI	systemic vascular resistance index
USA	United States of America
